# Imperforate Hyman

**Published:** 1896-11

**Authors:** J. Llewellyn Jones

**Affiliations:** Gatesville, Texas


For the Texas Medical Journal.

### IMPERFORATE HYMEN.

DR. J. LLEWELLYN JONES, GATESVILLE, TEXAS.

MY sole excuse for reporting this case is the fact that such cases, if not rare, are at least interesting, and show how important it is for the physician to insist on a further physical examination when he is in doubt as to his diagnosis, and not put off the examination until all remedies have failed, as is only too often the case with us.

On the night of October 11th, 1896, I was called to see Miss S., aged 16 years. Found her suffering intensely with coliky pains in lower part of the abdomen. Obtained the following history:

Is an orphan; has no mother; had lived with her father and step-mother in the Indian Territory for the past 13 years; had to work very hard, both in the house and in the field, and had finally left home and come to Texas a few days before I saw her. She had evidently been much neglected. Said she had suffered with similar pains for a few days each month for a year and a half, although there had never been any sign of blood. She was slightly swollen, and her abdomen became more full at each monthly period, and gradually reduced during the interim. With the exception of a few scattering sores on the face, she appeared to be in good health.

After explaining that a vaginal examination would be necessary, I proceeded to investigate, and on separating the labiæ found a baggy tumor of slightly pinky-bluish color, almost protruding from the vulva. Diagnosed imperforate hymen. Having explained the nature of the case to her friends, I made an incision in the hymen of about one-third or one half of an inch. A black, tarry fluid gushed out, saturated the cloths spread ready to receive it, overflowed these and gravitated into the bed.

Being afraid the evacuation of so large an amount of fluid at once might occasion shock, I occluded the opening for a few moments, but finding nothing more to follow but complete relief, allowed the balance to flow unchecked. This was in the early part of the night, and the flow continued more or less during the entire night. There was not less than half a gallon of fluid released at the first gush. The imprisoned fluid was not offensive. It being night I could not determine its exact color and consistence. After the vagina had been evacuated, a further examination showed the parts normal in other respects. An antiseptic wash was the only other treatment found necessary, and there has been no recurrence of the pains.

